# Case Report: Novel *ATP13A2* pathogenic variants associated with early-onset parkinsonism and a mini-review

**DOI:** 10.3389/fgene.2025.1588812

**Published:** 2025-07-29

**Authors:** Leonardo Affronte, Antonella Pini, Claudia Pizzoli, Emanuele Coccia, Serena Mazzone, Arber Golemi, Melania Giannotta, Duccio Maria Cordelli, Valerio Carelli, Alessandro Vaisfeld, Flavia Palombo

**Affiliations:** ^1^Department of Medical and Surgical Sciences (DIMEC), Alma Mater Studiorum, University of Bologna, Bologna, Italy; ^2^IRCCS Istituto delle Scienze Neurologiche di Bologna, Pediatric Neuromuscular Unit, UO Neuropsichiatria dell’Età Pediatrica, Bologna, Italy; ^3^IRCCS Istituto Delle Scienze Neurologiche di Bologna, UOC Neuropsichiatria dell'Età Pediatrica, Bologna, Italy; ^4^Medical Genetics Unit, IRCCS Azienda Ospedaliero-Universitaria di Bologna, Bologna, Italy; ^5^Medicina Nucleare, IRCCS Azienda Ospedaliero-Universitaria di Bologna, Bologna, Italy; ^6^Department of Biomedical and Neuromotor Sciences (DIBINEM), University of Bologna, Bologna, Italy; ^7^IRCCS Istituto delle Scienze Neurologiche di Bologna, Programma di Neurogenetica, Bologna, Italy

**Keywords:** ATP13A2, WES, juvenile onset parkinsonism, kufor rakeb syndrome, KRS cases revision

## Abstract

ATP13A2 is a gene localized on chromosome 1p36.13 and coding for a transmembrane protein found in the lysosomes and late endosomes, which is involved in many cellular metabolic activities. Pathogenetic variants of *ATP13A2* are associated with a wide range of neurodegenerative disorder including Kufor Rakeb syndrome (KRS), a rare autosomal recessive form of levodopa responsive juvenile onset parkinsonism (MxMD-*ATP13A2*), characterized by rapidly progressive muscular stiffness, bradykinesia, spasticity, pyramidal findings, dementia and supranuclear gaze palsy. The aim of this study is to provide detailed clinical descriptions of two siblings, carriers of novel biallelic *ATP13A2* variants. One of them showed KRS levodopa-responsive motor dystonic features at the age of 10 years preceded by moderate cognitive impairment, while the other only showed mild cognitive impairment at our last evaluation at 11 years of age. Additionally, we reviewed the previously published cases, focusing on early signs and symptoms, clinical evolution and response to therapy. To our knowledge, this is the only work that groups all reported KRS patients and compares their clinical and molecular features.

## Introduction

*ATP13A2* is a gene localized on chromosome 1p36.13 and coding for a transmembrane protein found in the lysosomes and late endosomes. The protein belongs to a P-type ATPases family whose role is to transport substrates through membranes by ATP hydrolysis and whose alterations underlie impairment in the metal/cation complex and mitochondrial homeostasis as well as lysosomal function (Martin et al., 2015; Sørensen et al., 2018; Van Veen et al., 2014). *ATP13A2* variants are associated with a wide range of neurodevelopment and neurodegenerative disorders including Kufor Rakeb syndrome (KRS), early-onset parkinsonism (EOPD), neuronal ceroid lipofuscinosis (NCL), hereditary spastic paraplegia (HSP) and amyotrophic lateral sclerosis-like form (Estrada-Cuzcano et al., 2017; Yahya et al., 2023). KRS is a rare autosomal recessive form of levodopa-responsive juvenile onset parkinsonism, previously known as PARK9 and now as MxMD-*ATP13A2* (Lange et al., 2022), that was initially described in 1994 in five members of a large family who lived in the Jordanian town of Kufor Rakeb, after which the disease was named (Najim al-Din et al., 1994; Brüggemann et al., 2010). In 2006 a large Chilean family with early-onset parkinsonism, whose characteristics resembled those of the Jordanian family, was reported and the *ATP13A2* gene was firstly associated to the syndrome (Ramirez et al., 2006). To confirm this association, *ATP13A2* pathogenic variants were investigated and found in the members of the original family (Ramirez et al., 2006).

KRS is characterized by early onset parkinsonism, usually between 12 and 16 years, and a rapid progression of clinical signs and symptoms. Motor manifestations include extrapyramidal findings such as rigidity, tremor, bradykinesia, postural instability with festinating gait, pyramidal findings such as spasticity with muscular stiffness, myoclonus, hyperreflexia and in some cases positive Babinski’s sign (Najim al-Din et al., 1994; Williams et al., 2005; Ramirez et al., 2006; Brüggemann et al., 2010). Additional motor signs include dystonia, ataxia, dyskinesia like facial-faucial-finger mini-myoclonus, dysarthria, dysphagia, slowed vertical and/or horizontal saccade eye movement and supranuclear upgaze palsy. Non-motor findings include cognitive decline that leads in some cases to dementia, visual hallucination and autonomic disorders such as urinary and fecal incontinence (Schneider et al., 2010; Yang and Xu, 2014). Only in a few cases the motor symptoms are reported to be preceded by cognitive impairment (Schneider et al., 2010; Crosiers et al., 2011; Niemann and Jankovic, 2019). In children, features such as psychomotor delay, early cognitive decline, pyramidal signs, ataxia and atypical movement disorders such as dystonia or choreoathetosis could make the diagnosis even more complicated (Paviour et al., 2004; Schrag and Schott, 2006; Garcia-Cazorla and Duarte, 2014; Niemann and Jankovic, 2019). This wide variety of signs and symptoms is probably due to a widespread brain involvement. KRS patients were examined with magnetic resonance imaging (MRI): common findings include diffuse brain atrophy and bilateral abnormal hypodensity on the T2 images in the putamen and caudate nuclei, due to iron accumulation (Brüggemann et al., 2010; Schneider et al., 2010; Yang and Xu, 2014). Moreover, in some patients the dopamine transporter (DAT) images revealed striatal tracer uptake under physiological levels (Brüggemann et al., 2010).

At present, there is no cure for KRS and the treatment is symptomatic. The major symptoms are sufficiently well controlled for some years by levodopa and carbidopa in most cases, while other medications such as dopamine agonists or trihexyphenidyl are reported as beneficial in some patients (Kola et al., 2022). Psychiatric symptoms, if they occur, can be controlled with antipsychotic drugs (McNeil-Gauthier et al., 2019). Second generation antipsychotic drugs should be used in order to reduce the occurrence of extrapyramidal side effects. Finally, deep brain stimulation can be considered as a treatment in the advanced cases (Kola et al., 2022).

The purpose of this work is to describe the phenotype of two siblings carrying the same novel pathogenic variants: a boy presenting with the typical phenotype at the age of 10 years, and his younger sister with only neuropsychological impairment. Furthermore, we provide a review of all cases published to date, with a focus on molecular data and phenotypic implications.

## Case 1 description

Patient 1 is a 14-year-old Italian boy, born at term by a normal delivery after a non-complicated pregnancy. His parents are not consanguineous and there is no history of neurological or psychiatric pathologies in the family. He had a mild motor milestone delay, starting to walk independently at around 2 years of age. He began to use single words and then to talk at standard age, having some phonological issues for which he was treated by a speech therapist for a few years, with a good response. During primary school he showed learning difficulties and at 7 years the Wechsler Intelligence Scale for Children–Fourth (WISC IV) was performed, showing a mild to moderate cognitive deficit (VCI 64, PRI 69, WMI 58, PSI 65, Full Scale IQ 53). At the age of 8 years he came for the first time to our attention. Neurological examination was normal, he was able to walk and run fast with no coordination problems and he was independent in everyday activities. A wakeful and sleep EEG showed occasional sharp waves in the fronto-central regions of the left hemisphere, not increased with sleep. EEG was repeated, but the previously observed abnormalities were no longer detected.

At about 10 years of age he became slower and showed an overall flexed posture. Neurological examination revealed a dystonic and flexed posture of the left arm and bilateral tremor of the hands. Urinary incontinence was reported. Strength and reflexes were normal. In a few months his condition worsened and amimia, facial-faucial-finger mini-myoclonus, reluctant speech, slowed vertical and horizontal saccade eye movement appeared. Moreover, worsening of cognitive ability was observed: WISC IV scale was performed at 11 years old (VCI 64, PRI 65, WMI 46, PSI 53, Full Scale IQ 44). This new result showed that those tasks evaluating the efficiency of cognitive processes were mainly involved, whereas pure verbal and non-verbal reasoning skills remained stable. Therefore, it seems that the functions worsening were verbal working memory (VMI) and executive speed tasks (PSI).

Routine blood tests and motor and sensory nerve conduction, showed no alteration. Brain and spinal cord MRI at the age of 11 was normal.

Whole exome sequencing (WES) was performed, showing compound heterozygosity for *ATP13A2* (MxMD-*ATP13A2*) variants (NM_022089.4): the c.2425dup variant, never reported and maternally inherited, is classified as likely pathogenic (PVS1, PM2_SUP), causing the misalignment of the reading frame with production of a truncated p. Ala809GlysTer49 protein; the c.3153dup variant, rare (2/1,614,040 alleles in gnomAD) and paternally inherited, is classified as pathogenic (PVS1, PM2_SUP), also causing a misalignment of the reading frame with production of a truncated protein p. Ser1052LeufsTer62. Moreover, the c.3153dup is already reported in ClinVar as a Pathogenic variant (VCV001968613.4). Pathogenic and likely pathogenic variants in other genes related to parkinsonism, to childhood onset dystonia, chorea or related movement disorder and to intellectual disability were excluded. Both *ATP13A2* variants were validated through Sanger sequencing. Parents did not show learning disability and were intellectually normal.

**FIGURE 1:**
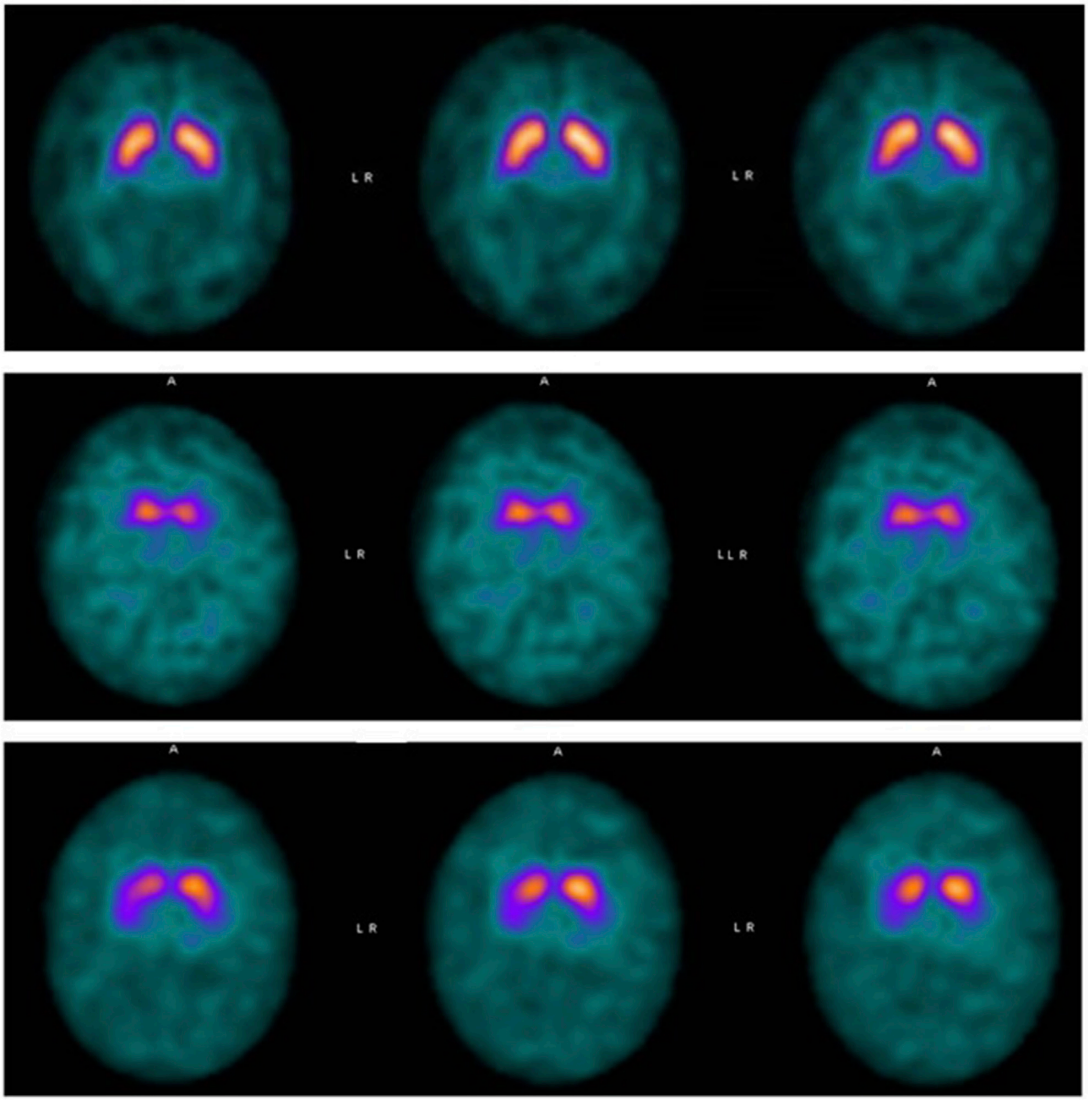
Top panel: DaTSCAN SPECT normal. Middle panel: Patient 1: ligand binding in both putamina was almost absent. The uptake was also markedly and symmetrically reduced in the caudate nucleus bilaterally. Bottom panel: Patient 2: DaTSCAN SPECT showed considerably decreased ligand binding in both putamina. Initial uptake reduction was observed also in the right caudate.

After the diagnosis a dopamine transporter scan (DaTscan) was performed, revealing bilaterally reduced availability of the presynaptic dopamine transporters in the putamen and a mild bilateral uptake reduction in the caudate nucleus compared with normal controls. These findings represent a bilateral and symmetric impairment of the nigro-striatal system (Figure 1).

The autonomic control of the cardiovascular system was studied by deep-breathing test and tilt test, both of which were normal for age.

The child was treated from the age of 12 years with levodopa, with benefits. Clinically, movements became more fluid and rapid, gait skills improved, the face was more expressive, he regained sphincteric control and his speech was also more comprehensible. Moreover, a scopolamine patch was used to control sialorrhea. No collateral effects related to levodopa have been observed so far, at 14 years of age. The cognitive profile on the WISC IV scale at the age of 13 resulted in VCI 58, PRI 56, WMI 46, PSI 47, and Full Scale IQ 40; a reevaluation at age 14 years and 8 months showed improvement in visual logical reasoning skills and poor development in processing speed and verbal memory (VCI 52, PRI 63, WMI 46, PSI 47 Full scale IQ 40). Auditory attention in a signal detection task also proved to be in the expected average for age (subtest Auditory Attention Nepsy 2 50th for age). This task was not administered the previous year due to difficulty in maintaining delivery and number of false recognitions exceeding correct responses.

## Case 2 description

The sister of patient 1 came to our attention at the age of 11 years following her brother’s diagnosis and carried the same genetic diagnosis.

Her neurological examination was normal. She appeared as a quiet girl, her father reported adequate social and relational skills. The neuropsychological evaluation showed general cognitive skills below average, assessed by the WISC-IV test (VCI 80, PRI 78, WMI 76, PSI 65, Full Scale IQ 67). The profile was homogeneous, apart from the visuo-graphic processing speed index. This score is influenced by the specific subitem “Coding” of WISC-IV, the graphic speed test, which shows a lower result than the other tests, as if to indicate a greater involvement of fine motor skills. Planning and visuomotor integration skills were also investigated by VMI test, showing a lower score than the average for age (VMI, 8th percentile). It therefore seemed that motor action planning was the first and most compromised domain. Awake EEG was normal. Brain DaTSCAN SPECT showed considerably decreased ligand binding in both putamina (Figure 1). Initial uptake reduction was observed also in the right caudate. Vegetative system function was also studied in this patient. The autonomic control of the cardiovascular system was assessed by performing deep-breathing test, tilt test, and Valsalva maneuver, which were normal for age.

The girl is currently not treated with any medication and attends school with support.

## Discussion

Parkinsonism is a frequent condition characterized by bradykinesia, rigidity, tremor and postural instability, most often affecting patients over 60 years of age. Only around 5% of the patients are less than 50 years old (Niemann and Jankovic, 2019). The population with early-onset parkinsonism (EOP) is further arbitrarily subdivided by age of onset. Individuals whose symptoms begin between the ages of 21 and 50 are categorized under the term “*young onset parkinsonism*” (YOP). However, if the neurological symptoms start before 20 years of age the syndrome is called “*juvenile parkinsonism*” (JP) (Paviour et al., 2004; Garcia-Cazorla and Duarte, 2014; Niemann and Jankovic, 2019). From an etiological point of view, JP may be the results of acquired or genetic causes. The first group includes some of the most common causes including drug side effects, structural brain lesions, such as hypoxic-ischemic encephalopathy or basal ganglia tumors, encephalitis and immunomediated disease. The second group could be split up into inborn errors of metabolism (IEM) or other genetic causes different from IEM (Garcia-Cazorla and Duarte, 2014). KRS is classified in the JP group and is known for being an autosomal recessive disease that causes iron accumulation in the brain, thus also classified as IEM (Schneider et al., 2010; Garcia-Cazorla and Duarte, 2014; Estrada-Cuzcano et al., 2017).

**FIGURE 2:**
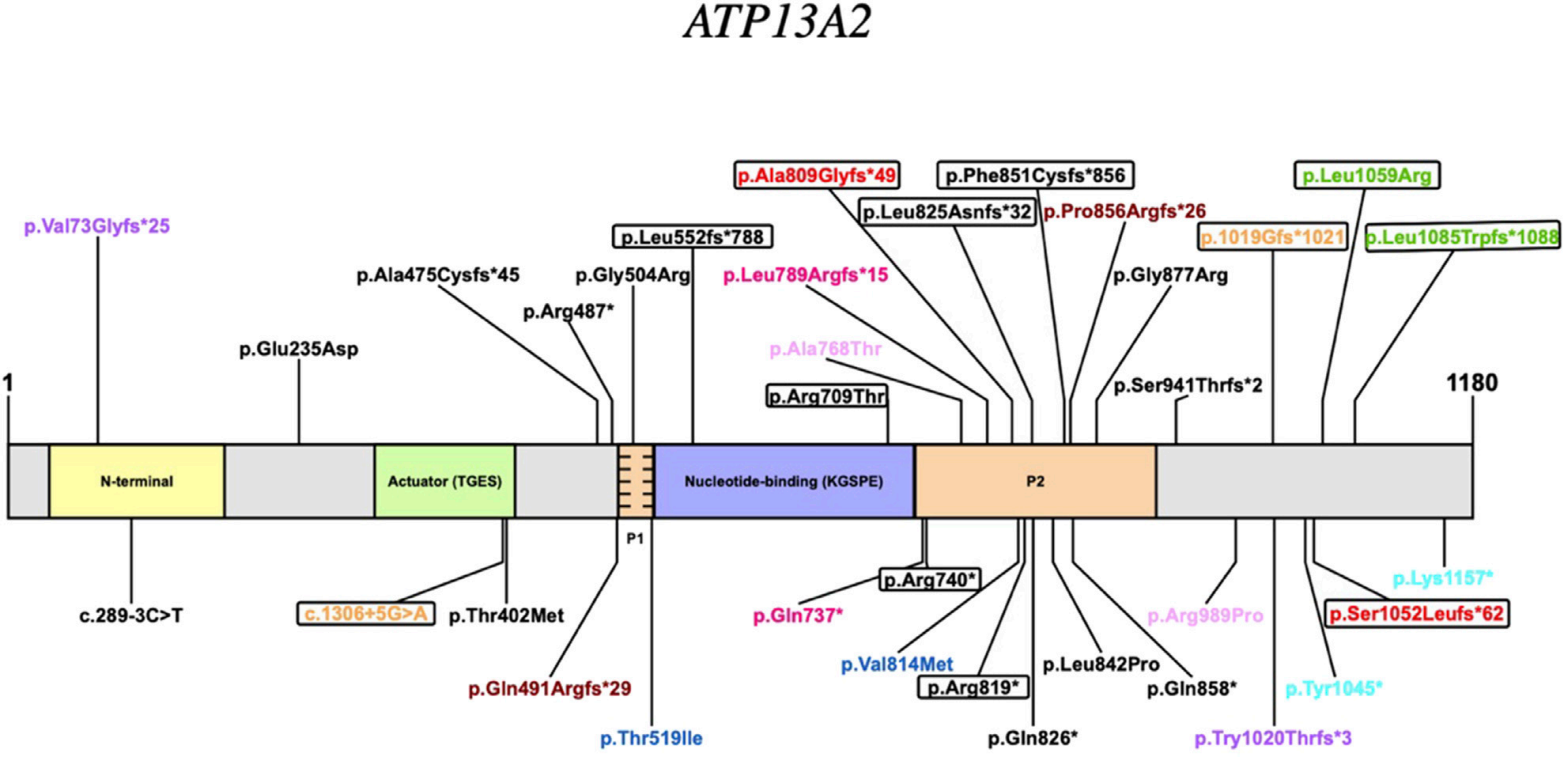
Pathogenic variants of the *ATP13A2* gene. Homozygous variants are depicted in black; compound heterozygous variants share the same colour (variants of our cases are in red); encircled variants are reported in patients who presented with “DD developmental delay” or “CD cognitive deficit” as initial symptoms.

KRS is an extremely rare disease and this emerges from the small number of cases reported in the literature. Possibly, this is also due to the recent identification of the genetic basis for this disorder. Thus, we reviewed all published cases and to our knowledge, this is the first analysis that groups all reported KRS patients comparing their clinical and molecular features (Table 1). Variants are listed according to the position on the of ATP13A2 protein (Figure 2). We also summarize 15 patients harboring heterozygous pathogenic *ATP13A2* variants, who have received a clinical diagnosis of EOPD (Supplementary Table S1) (Di Fonzo et al., 2007; Lin et al., 2008; Djarmati et al., 2009; Chen et al., 2011; Fong et al., 2011; Wang et al., 2020). Clinical and neuroimaging data of KRS patients are summarized in Table 2. Our patients presented with symptoms that are partly characteristic of KRS and partly distinctive.

**TABLE 1:** Patients with a clinical diagnosis of KRS.

Ref	Mutation	S	AO	IS	ES	P	O	C	OF	Imaging	LR
Ramirez et al. (2006)	c.1632_1653dup22 (p.Leu552fsX788) - HO	M	12	B, CD, R	B, G, PI, R	BTS, MC, Sp	SH, SUP, SV	NO	FFF, Si, VH	DA (MRI)	+
		M	13	B, R	B, MD, PI, R	BTS, MC, Sp	SH, SUP, SV	CD	DY, FFF, Si, VH	DA (MRI)	+
		M	12	CD, R	B, MD, PI, R	BTS, MC, Sp	SUP, SV	Mild CD	DY, FFF, Si, VH	Mild DA (MRI)	+
		F	12	B	B, G, MD, PI, R	BTS, MC, Sp	SUP, SV	NO	FFF, Si, VH	NR	+
Behrens et al. (2010)	c.3057delC (p.1019GfsX1021) c.1306 + 5G>A- HEc	M	13	F, LD	B, R, T	BS, H, Sp	SUP	NR	FFF, Insomnia, Perinatal ischemia, VH	Enlarged sulci (CT)	Not tried
		M	12	LD, B, R	B, R	BS, Sp	SH, SUP, SV	MMSE 19/30	Auditory hallucinations, FFF, Insomnia	Mild DA, Caudate hypointensity (MRI)	-
		M	10	B, F, LD	B, R, T	Bs, H, Sp	NO	NR	FFF, Insomnia	NR	-
		F	10	F, LD	B, R	BS, H, MC	SH, SUP, SV	MMSE 15/30	FFF, Insomnia	DA (CT)	-
		M	12	LD	B, R	BS, H, MC, Sp	SUP, SV	MMSE 9/28	FFF, Insomnia	NA	Not tried
Schneider et al. (2010)	c.1510G>C (Gly504Arg) HO	M	12	B	B, MD, PI, R	BTS, H, MC	SUP	MMSE 29/30	PE, VH	DA (CT)	+
Santoro et al. (2011)	c.2629G>A (p.Gly877Arg) - HO	M	10	B, G	B, MD	BS, BTS, H, MC	SH, SUP, SV	CD	DY, FFF	DA (MRI), nigrostriatal dopaminergic defects (DaTScan SPECT)	+
Crosiers et al. (2011)	c.2742_2743delTT (p.F851CfsX856) - HO	M	10	B, CD, T	B, MD, R, T	BTS	SV	IQt 45 (WISC III)	Scoliosis, PE, VH	NO (MRI), Putamen and Caudate decreased ligand binding (^123^I-FP-CIT SPECT)	+
Park et al. (2011)	c.3176T>G (p.Leu1059Arg) c.3253delC (p.Leu1085TrpfsX1088) - HEc	M	17	DD, Social Anxiety	B, PI, R, T	BTS	SH, SUP, SV	CD	FFF, Olfactory impairment, PE	NO (MRI)	+
		F	17	Anxiety	B, MD, G, R, T	BTS	SH, SUP, SV	CD	FFF, PE	NO (MRI)	+
Eiberg et al. (2012)	c.2473C>AA, (p.Leu825AsnfsX32) HO	F	27	Fatigue, PI	NR	BS, BTS, H	NR	CD	NR	DA (CT)	NR
		M	24	Diplopia, Weakness	NR	NR	Diplopia	CD	Auditory hallucinations, PE	DA (MRI)	NR
		M	12	SI, T, retrocollis	B, R	BS, BTS, H, MC	SUP	CD	FFF, Limb ataxia, Auditory and VH	Nonspecific hyperintensity (MRI), Reduced uptake in striatum (DaTscan)	NR
		F	10	CD	B, R	NO	NO	CD	NR	NO (CT), Reduced uptake in striatum (DaTscan)	NR
		F	29	G	NO	BS, BTS, H, MC	SH, SUP, SV	Probably CD	FFF, Truncal and limb ataxia	NO (CT), Enlarged ventricles and gracile spinal cord (MRI)	NR
		F	15	CD	B, R	NR	NR	CD	NR	NO (CD)	NR
Malakouti-Nejad et al. (2014)	c.2762C>T (p.Gln858X) HO	F	14	Motor defect	B, MD, PI, R, T	BS	SH, SV	LD	Ataxia, DY, FFF	Cerebellar atrophy (MRI)	+
		M	10	B	B, MD, PI, R, T	BS	SH, SUP, SV	CD	Ataxia, DY, FFF	Cerebellar atrophy (MRI)	+
		M	30	NR	B, MD, PI, R, T	NR	NR	NR	DY	NR	NR
Martino et al. (2015)	c.2822delG (p.Ser941Tfs1X) - HO	M	14	MD	B, G, MD, PI, R	BTS, H, Sp	SUP, SV	NO	DY, FFF	Bilateral reduced putamen uptake (DaTscan), DA and iron accumulation (MRI)	+
Prashanth et al. (2015)	c.2476C>T (p.Gln826X) HO	M	16	Isolation	B, MD, R, T	NO	SH, SV	NR	Anxiety	NO (MRI)	+
Rohani et al. (2017)	c.2455C>T (p.Arg819X) HO	F	10	B	B, PI, R, T	BTS, BS, Sp	SH, SUP, SV	Severe CD	Action myoclonus, Ataxia, Depression, FFF, Pes cavus, I, VH	DA (MRI)	+
		F	10	Isolation, Seizure	B, G, PI, R, T	BTS, BS, Sp	SH, SUP, SV	Prominent CD	FFF, Freezing of gait, Pes cavus, I	NR	+
		F	12	DD, Seizure	B, G, PI, R, T	BTS, Sp	SH, SUP, SV	Mild CD	Ataxia, FFF, Pes cavus	NO (MRI)	+
		M	11	B	B, G, PI, R, T	BTS, BS, Sp	SH, SUP, SV	Mild CD	Depression, FFF, VH, Si	NO (MRI)	+
		F	12	B	B, G, PI, R, T	BTS, Sp	SH, SUP, SV	Mild CD	Depression, FFF, Si	NR	+
Jain et al. (2016)	NR	M	24	Abnormal behavior	B, MD, T	BTS, BS, MC, Sp	SUP	CD	FFF	“eye of tiger” sign (MRI)	-
Abbas et al. (2016)	c.2525T>C (p.Leu842Pro) - HO	M	21	B	B, MD, R, T	BTS, Sp	SH, SUP, SV	MMSE 8/30	Auditory and VH, I, PE, Hypersexuality	DA (MRI)	+
Inzelberg et al. (2018)	c.3057delC (p.Try1020ThrfsX3) c.217_218insG (p.Val73GlyfsX25)- HEc	M	27	G	B, R	BS, BTS	SH, SUP, SV	Mild CD	Ataxia, DY, VH	DA (MRI), Reduced caudate and putamen uptake (DaTscan)	+
		F	23	Seizure	B, R	BS, BTS	SH, SUP, SV	NO	Ataxia, DY, Emotional lability	DA (MRI), Reduced caudate and putamen uptake (DaTscan)	Not tried
Noch et al. (2017)	c.1459C>T (p.Arg487X) HO	M	8	LD	B, G, R	BTS	SUP	CD	DY	Dysplasia of corpus callosum’s rostrum	+
		M	13	DY	B, G, PI, R	BS, BTS	SUP	MoCA 24/30	Ataxia, DY	DA (MRI)	+
McNeil-Gauthier et al. (2019)	c.2126G>C (p.Arg709Thr) - HO	M	NR	DD	B, T	Sp	SUP	CD	Aggressive behavioral, Depression, DY, VH	DA (MRI)	+
Gowda et al. (2020)	c.1556C>T (p.Thr519lle) c.2440G>A (p.Val814Met) - HEc	M	2	NR	Parkinsonism	Pyramidal tract dysfunction	SUP	CD	Behavioral problems, Oculogyric crisis	NO (MRI)	+
Pietrzak et al. (2019)	c.2366_2367delTC (p.Leu789ArgfsX15) c.2209C>T, (p.Gln737X) HEc	F	1	Spastic tetraplegia	B, G, MD, PI, R, T	Sp	SH, SUP, SV	Severe CD	Action myoclonus, Ataxia, DY, PE, Si	DA (MRI)	+
De Michele et al. (2020)	c.1205C>T (p.Thr402Met) - HO	M	38	Ataxia	DYS	BTS, MC	NO	NO	Action myoclonus	Mild DA (MRI)	NR
Balint et al. (2020)	c.2218C>T (p.Arg740Ter) - HO	M	NR	DD	MD, PI	BTS, Spastic G	SUP	CD	Autistic spectrum, Gastroesophageal reflux, Hypospadia, Laryngomalacia	NO (MRI), bilaterally reduced availability of the presynaptic dopamine transporters (DaTscan)	+
	c. 1472_1473del (p.Gln491ArgfsX29) c.2567_2568del (p.Pro856ArgfsX26) HEc	F	24	Paranoid ideas	B, G, T	BTS, MC, Sp	SUP	CD	Bulimia nervosa, DY, PE, VH	DA and “ear of the lynx” sign (MRI), asymmetric reduction (worse on left) of dopamine uptake (DaTscan)	+
Manrique et al. (2021)	c.3135C>A; (p.Tyr1045X) c.3469A>T (p.Lys1157X) - HEc	M	39	Action myoclonus, Ataxia	NR	BTS, MC	SH, SV	Mild CD	Ataxia, DY, FFF, Pes cavus	DA (MRI)	NR
Kırımtay et al. (2021)	c.1422_1423del (p.Ala475CysfsX45) - HO	M	10	Speech disorder	B, G, R	BTS, BS	SUP	IQ 79 (?)	Aggressive behavior, DY, Si	Severe DA and iron accumulation (MRI)	-
		F	15	LD	B, G, PI	BTS, BS	NR	IQ 71 (?)	DY, FFF, Hair loss, Risussadonicus, Scoliosis, Si	DA (MRI)	-
Kaul et al. (2016)	NR	M	NR	NR	B, G, PI	NR	NR	NR	Ataxia, DY, Impaired fine motor skills, Micrographia	NR	NR
		M	NR	NR	B, G, PI	NR	NR	NR	Ataxia, DY, Impaired fine motor skills, Micrographia	NR	NR
Kola et al. (2022)	c289-3C>T - HO	M	31	T	B, G, MD, R, T	BS, BTS	SH, SV	MoCA 7/30	NR	DA (MRI)	+
Satolli et al. (2023)	c.2966G>C (p.Arg989Pro) c.2302G>C (p.Ala768Thr) HEc	F	11	MD, R, T	MD, R, T	NO	SH, SV	Mild CD	Depression, FFF, Hypersexuality, VH	DA and iron accumulation (MRI), Reduced putamen uptake (DaTscan)	+
Gurram et al. (2023)	c.705G>C (p.Glu235Asp) HO	M	16	DY, Oculogyriccrisis	B, MD, R	H	SUP	NO	DY, Oculogyriccrisis	NO (MRI)	+
Patient 1 and 2	c.2425dup (p.Ala809GlyfsX49) c.3153dup (p.Ser1052LeufsX62) HEc	M	2	DD	B, G, MD, R, T	BTS, MC	SH, SUP, SV	CD	DY, FFF, Si, I	NO (MRI), Reduced caudate and putamen uptake (DaTscan)	+
		F	11	LD	NO	NO	NO	Mild CD	NO	Reduced caudate and putamen uptake (DaTscan)	NR

AO, age of onset (years); B, bradykinesia; BS, Babinski’s sign; BTR, brisk tendon reflex; C, cognitive; CD, cognitive deficit; DA, diffuse atrophy; DD, development delay; DY, dysarthria and dysphagia; Es, extrapyramidal signs and symptoms; F, fatigue; FFF, facial-faucial-finger mini-myoclonus; G, gait disturbance; H, hyperreflexia; HEc, compound heterozygosity; HO, homozygous; I, incontinence (urinary and/or fecal); IS, initial symptoms; LD, learning difficulties; LR, levodopa response; MC, myoclonus; MD, movement disorder (dystonia, dyskinesia); NO, normal/not observed; NR, non reported; O, oculomotion; OF, other finding; OP, our patient; P, pyramidal signs and symptoms; PE, psychotic episodes; PI, postural instability; PN, patient number, R, rigidity; S, sex; Si, sialorrhea; SH, slowed horizontal saccade eye movement; Sp, spasticity; SUP, supranuclear up-gaze palsy; SV, slowed vertical saccade eye movement; T, tremor; VH, visual hallucination.

**TABLE 2:** Summary of clinical and neuroimaging data of KRS.

		KRS patients (52, Table1)
Gender		Male = 34/52 (65.38%) Female = 18/52 (34.62%)
Onset (years)		15.5 y (1y-39y) on 48 patients
Signs and symptoms (number of patients and percentage)	Initial	Es = 16 (30.77%) Mix (signs and symptoms of different categories) = 14 (26.92%) LD = 4 (7.69%) NR = 4 (7.69%) DD = 3 (5.77%) CD = 2 (3.85%) Seizure = 1 (1.92%) P = 1 (1.92%) Anxiety = 1 (1.92%) Isolation = 1 (1.92%) Paranoid ideas = 1 (1.92%) Speech disorder = 1 (1.92%) Speech delay = 1 (1.92%) Abnormal behavior = 1 (1.92%) DY = 1 (1.92%) Ataxia = 1 (1.92%)
	Extrapyramidal	Reported = 47 (90.38%) NR = 3 (5.77%) NO = 2 (7.69%)
	Pyramidal	Reported = 44 (84.61%) NO = 4 (7.69%) NR = 4 (7.69%)
	Ocular	Reported = 41 (78.84%) NR = 7 (13.46%) NO = 4 (7.69%)
	Cognitive deficit	Reported = 41 (78.84%) NO = 5 (9.62%) NR = 6 (11.54%)
	Others	FFF = 26 (50%) Hallucination (visual and/or auditory) = 18 (34.62%) DY = 16 (30.77%) Ataxia = 13 (25%) Si = 11 (21.15%) PE = 7 (13.46%) NR = 6 (11.53%) Depression = 5 (9.61%) Pes cavus = 4 (7.69%) I = 4 (7.69%) Aggressive behavior = 3 (5.77%) Action myoclonus = 3 (5.77%) Scoliosis = 2 (3.85%) Hypersexuality = 2 (3.85%) Perinatal ischemia = 1 (1.92%) Olfactory impairment = 1 (1.92%) Anxiety = 1 (1.92%) Hair loss = 1 (1.92%) Risus sardonicus = 1 (1.92%) Emotional lability = 1 (1.92%) Oculogyric crisis = 1 (1.92%) Autism spectrum disorder = 1 (1.92%) Gastrointestinalreflux = 1 (1.92%) Hypospadia = 1 (1.92%) Laryngomalacia = 1 (1.92%) Bulimia nervosa = 1 (1.92%) Oculogyriccrisis = 1 (1.92%) Micrographia = 1 (1.92%)
Neuroimaging findings		DA = 23 (44.23%) NO = 13 (25%) SPECT abnormalities = 10 (19.23%) NR = 7 (13.46%) Other = 6 (11.54%) Iron accumulation = 3 (5.77%)
LR		Positive (+) = 31 (59.62%) NR = 12 (23.07%) Negative (−) = 6 (11.54%) Not Treated = 3 (5.77%)

CD, cognitive deficit; DA, diffuse atrophy; DD, development delay; DYS, dysarthria and dysphagia; Es, extrapyramidal signs and symptoms; FFF, facial-faucial-finger mini-myoclonus; I, incontinence (urinary and/or fecal), LD, learning difficulties; LR, levodopa response; MD, movement disorder (dystonia, dyskinesia), NO, normal/not observed; NR, non reported; OF, other finding; OP, our patient; P, pyramidal signs and symptoms; PE, psychotic episodes; Si, sialorrhea.

A gender bias is observed in KRS patients, being the male-to-female ratio almost 2:1 (Table 2). Concerning the age of onset, mean age is 15.5 years, with a wide range, spanning from a minimum of 1 year to a maximum of 39 years (Table 2), (Ramirez et al., 2006; Rohani et al., 2017; Niemann and Jankovich.,2019). According with literature data, our patients present an early onset at 2 and 11 years, respectively. However, the initial symptoms observed in our patients are remarkable compared with the reported KRS population: the developmental delay observed in patient 1 has been described in only 2 other cases (5.77%) and the learning difficulties observed in patient 2 have been reported in 3 other cases (7.69%). Patient 1 showed a rapidly progressive disease course as most reported patients (Ramirez et al., 2006; Kirimtay et al., 2021; Satolli et al., 2023). The presence of extrapyramidal signs and symptoms, observed in 90.38% of the KRS population, and pyramidal signs and symptoms, described in 84.61% of the KRS population, are observed in patient 1 but not yet in patient 2, at the age of 11 years. Ocular movements impairment, present in patient 1, is reported in the majority of KRS cases (78.84%). The cognitive decline shown by our patient 1 is a very frequent symptom among KRS patients (78.84%). Many other signs and symptoms can be found in KRS: amongst them, facial-finger mini myoclonus, dysphagia, sialorrhea and urinary incontinence. Those are all present in our patient 1. They are some of the most frequent additional findings in KRS, with a recurrence rate of 50%, 30.77%, 21.15% and 7.69%, respectively.

MRI images showed no abnormality in patient 1, corresponding to the second most observed finding in KRS (25%). The young age of patient 1 and the early execution of the MRI are the factors that probably determined the normal result of the examination, which is generally altered in patients with a longer disease course. Moreover, as for some other patients, [123I]FP-CIT–SPECT showed in both our siblings a decrease of dopamine transporter (DaT) density in the striatum that corresponds to a neuronal loss in this brain area. This finding has been observed in other 8 KRS patients (19.23% of the total). A good response to the symptomatic treatment by levodopa is an important element to highlight as the 59.62% of KRS patients respond positively, like in our patient 1. However, a high rate (23.07%) of “*not reported*” response must be considered.

It is tempting to speculate that the observed clinical differences in KRS patients could be explained by the type and position of *ATP13A2* variants. In general, there seems to be a predominance of Loss of Function (LoF) variants (24/35) (Figure 2). Although, it cannot be ruled out that missense variants may also have a LoF effect. Literature data from reported KRS patients suggests that the P2 domain could be a hotspot (Table 1). In fact, variants affecting this domain are numerous accounting for 40% of the total variants (14/35) with a total of 24 patients having at least one allele with a variant in this region. Some phenotypic similarities are observed among the patients in this group such as the age of the onset less than or equal to 11 years of age. Another common feature of the group is the cognitive deficit of variable severity appearing, as in Patient 1 and 2, at the onset of the disease in 5 other patients. From a therapeutic perspective, all patients in this group who were administered levodopa presented a positive response.

However, the two patients described in the present study display also peculiar features that allow some speculations about the brain involvement in KRS due to *ATP13A2* gene variants. Patient 1 presented a main involvement of the cognitive efficiency systems and a more severe intellectual disability than those reported in the literature. In patient 2 neuropsychological examination showed only movement planning and visuo-motor integration skills impairment. Therefore, both patients show cognitive impairment with different severity at onset, despite the absence of possible pathogenic variants in DD/ID genes that may act as modifiers. Given the functions involved, although different, there seems to be a primary involvement of the parietal-frontal areas.

In the families reported to date, the phenomenon of “*incomplete penetrance*”, i.e., cases with pathogenic variants and absence of symptoms, has never been reported. Patients with a late onset and/or slow progression, which can therefore be considered as paucisymptomatic, at least at the onset, are instead described (De Michele et al., 2020; Manrique et al., 2021). However, since they have no siblings, no comparisons could be made. In multi-member families the onset and clinical presentation are homogeneous, except for two families with a certain variability of symptom onset (e.g., from 10 to 29 years) (Eiberg et al., 2012; Malakouti-Nejad et al., 2014). However, once the symptoms have arisen, they rapidly evolved to a similar severity in all members. We also explored a gender-related difference of clinical expressivity and/or age of onset, which did not emerge in the families reported. Undoubtedly, the major bias in reporting a variable expressivity in siblings symptomatology is due to early diagnosis in younger one, as in our family, and this could explain the absence, so far, of the movement disorder phenotype in patient 2 which may eventually develop in the future.

The reasons behind the two unusual presentations are unclear, however we hypothesize that this may depend on a more extensive involvement of the brain, and not only of the basal ganglia. Remaining on the speculative ground also the motor functions of nigro-striatal circuits are affected later than cognitive functions in the presence of an ATP13A2-related cellular energy deficit. Further, we studied in both patients the vegetative system, which had never been done in *ATP13A2* patients, finding that was essentially unimpaired based on the tests performed.

Another possible cause of phenotypic variability could be the modifying effect of specific variants within the ATP13A2 protein, according to variant’s type and position. The P2 domain seems to be a hotspot, with a developmental delay severity that is apparently linked to specific variants. However, the small number of cases does not allow definitive conclusions. In addition, the fact that patients with the same *ATP13A2* variants experiences different disease onset and progression suggests that other unknown genetic factors may modify the overall effect.

## Conclusion

We report here novel pathogenic variants in the *ATP13A2* gene causing early onset Kufor Rabek Syndrome presenting with early onset cognitive impairment. To our knowledge patient 2 is the first case with genetic diagnosis characterized by cognitive impairment preceding the motor presentation characteristic of KRS displaying concomitant cognitive motor action planning impairment and learning disability. In this specific case, the diagnosis was due to the previously diagnosed brother. Due to the potential therapeutic approach and to better understand the disease course, we suggest that KRS should be considered even in atypical presentations with only cognitive impairment and *ATP13A2* should be included in the genes panel for cognitive delay.

## Data Availability

The data presented in the study are deposited in the BioProject database, accession number BioProject ID: PRJNA1214690.
