# Supplementary material for: Case Report: Novel ATP13A2 pathogenic variants associated with early-onset parkinsonism and a mini-review
Source: Front Genet. 2025 Jul 29;16:1588812. doi: 10.3389/fgene.2025.1588812 (PMC12340552; doi:10.3389/fgene.2025.1588812)
Supplement: Supplementary file 1 [file Table1.docx]

| **Ref** | **Mutation** | **S** | **AO** | **IS** | **Es** | **P** | **O** | **C** | **OF** | **Imaging** | **LR** |
| --- | --- | --- | --- | --- | --- | --- | --- | --- | --- | --- | --- |
| (Di Fonzo et al., 2007) | c.35C>T (Thr12Met)  HE | M | 30 | NR | B, R, T, | H | NO | NO | NO | NO (MRI) | + |
|  |  |  |  |  |  |  |  |  |  |  |  |
|  | c.1597C>A (Gly533Arg) HE | M | 40 | NR | B, R | H | NO | NO | PE, VH, Obsessive-compulsive disturbance | NO (MRI) | + |
| (Lin et al., 2008) | c.2236G>A (Ala746Thr) HE | F | 53 | NR | B, MD, R, T | BS | NO | NO | NR | NO (MRI) | + |
|  |  | M | 50 | NR | B, MD, PI, R, T | BS | NO | NO | NR | NO (MRI) | + |
|  |  | M | 39 | NR | B, MD, PI, R, T | BS | NO | NO | NR | NO (MRI) | + |
| (Djarmati et al., 2009) | c.746C>T (p.Ala249Val) HE | F | 31 | T | B, MD, PI, R, T, | NO | NO | NO | Depression | NO (MRI) | + |
|  | c.844A>T (p.Ser282Cys) HE | M | 20 | MD | B, MD, PI, R | NO | NO | NO | NO | NO (MRI) | + |
|  | c.2939G>A (p.Arg980His)- HE | M | 36 | T | B, MD, PI, R, T | NO | NO | NO | Depression | DA (CT) | + |
|  | c.1346G>A (p.Arg449Gln)- HE | F | 35 | T | B, MD, PI, R, T, | NO | Bilateral ptosis, SV | NO | Depression, DY, I | NO (MRI) | + |
| (Fong et al., 2011) | c.1108 1120del13 (p.Arg370fsX390) - HE | M | 5 | DYS, MD | B, MD, PI, R, T | NO | NO | NO | NO | NO (MRI), Reduction of activity in lentiform nuclei (^123^I-FP-CIT SPECT) | + |
| (Chen et al., 2011) | c.3274A>G (p.Gly1014Ser) - HE | F | 48 | NR | B, PI, R, T | NR | NR | NR | NR | NO (MRI) | + |
|  | c.2236G>A (Ala746Thr) HE | M | 49 | NR | B, PI, R, T | NR | NR | NR | NR | NR | + |
|  |  | F | 51 | NR | B, PI, R, T | NR | NR | NR | NR | NR | + |
| (Jain et al., 2016) | NR | M | 24 | Abnormal behavior | B, MD, T | BTS, BS, MC, Sp | SUP | CD | FFF | “eye of tiger” sign (MRI) | - |
| (Wang et al., 2020) | c.2236G>A (Ala746Thr) HE | F | 46 | NR | MD, PI, R, T | NR | NR | NR | NR | NR | - |

**Supplementary Table S1.** Patients with EOPD harboring an *ATP13A2* pathogenetic variant in heterozygosity

AO = age of onset (years), B: bradykinesia, BS = Babinski’s sign, BTR = brisk tendon reflex, C = cognitive, CD = cognitive deficit, DA = diffuse atrophy, DD = development delay, DY = dysarthria and dysphagia, Es = extrapyramidal signs and symptoms, F = fatigue, FFF = facial-faucial-finger mini-myoclonus, G = gait disturbance, H = hyperreflexia, HE = heterozygosity, I = incontinence (urinary and/or fecal), IS = initial symptoms, LD = learning difficulties, LR = levodopa response, MC = myoclonus, MD = movement disorder (dystonia, dyskinesia), NO = normal/not observed, NR = non reported, O = oculomotion, OF = other finding, OP = our patient, P = pyramidal signs and symptoms, PE = psychotic episodes, PI = postural instability, PN = patient number, R= rigidity, S = sex, Si = sialorrhea, SH = slowed horizontal saccade eye movement, Sp = spasticity, SUP =supranuclear up-gaze palsy, SV = slowed vertical saccade eye movement, T = tremor, VH = visual hallucination
